# Combined Sarcoidosis and Idiopathic Pulmonary Fibrosis (CSIPF): A New Phenotype or a Fortuitous Overlap? Scoping Review and Case Series

**DOI:** 10.3390/jcm11072065

**Published:** 2022-04-06

**Authors:** Laura Bergantini, Gabriele Nardelli, Miriana d’Alessandro, Giusy Montuori, Caterina Piccioli, Elisabetta Rosi, Sara Gangi, Dalila Cavallaro, Paolo Cameli, Elena Bargagli

**Affiliations:** 1 Respiratory Disease Unit, Department of Medical Sciences, Surgery and Neurosciences, Azienda Ospedaliera Universitaria Senese (AOUS), 53100 Siena, Italy; gabriele.nardelli@student.unisi.it (G.N.); dalessandro.miriana@gmail.com (M.d.); giusy.montuori97@gmail.com (G.M.); sara.gangi@student.unisi.it (S.G.); cavallarodalila@gmail.com (D.C.); paolocameli88@gmail.com (P.C.); bargagli2@gmail.com (E.B.); 2 SOD of Respiratory Diseases, Florence University Hospital, 50100 Florence, Italy; caterinapicc@gmail.com (C.P.); rosiel@aou-careggi.toscana.it (E.R.)

**Keywords:** CSIPF, lone-IPF, fibrotic sarcoidosis, scoping review

## Abstract

Idiopathic pulmonary fibrosis (IPF) and sarcoidosis are two distinct clinical entities with different aetiology, epidemiology, risk factors, symptoms and chest imaging. A number of papers have reported an overlap of the two diseases and have suggested the existence of a distinct phenotype defined as combined sarcoidosis and idiopathic pulmonary fibrosis (CSIPF). We used the scoping review protocol to review the literature on CSIPF. We also enrolled a cohort of nine CSIPF patients and compared them with lone-IPF and fibrotic sarcoidosis patients. Our CSIPF cohort showed male prevalence and only ex-smokers. Functional assessment at baseline showed mild to moderate restrictive impairment of lung volumes in lone-IPF and CSIPF patients, associated with moderate-to-severe reduction in DLco percentages. Although all CSIPF patients were on antifibrotic treatments, functional impairment occurred in the two years of follow up. This suggests the importance of considering these patients at high risk of rapid deterioration and lung damage.

## 1. Introduction

Interstitial lung diseases are a heterogenous group of more than 200 lung disorders characterized by inflamed interstitium and fibrosis of the parenchyma leading to thickening of the alveolar walls. All are associated with substantial morbidity and mortality [1,2,3,4]. Interstitial lung diseases are classified in five categories: idiopathic interstitial pneumonias (IIPs), autoimmune ILDs, hypersensitivity pneumonitis, sarcoidosis and other ILDs [5].

Idiopathic pulmonary fibrosis (IPF) and sarcoidosis are two distinct clinical entities. The former is a chronic fibrosing lung disease that is diagnosed in the absence of any identifiable underlying pathological/clinical cause. It typically affects adult males with unexplained chronic dyspnoea but no other symptoms [6,7,8]. Older age and smoking are considered major risk factors for the development of IPF [9,10]. Usual interstitial pneumonia (UIP) is the histopathological and HRCT hallmark of IPF [6,11], characterized by honeycombing and sometimes associated with mediastinal lymphadenopathy. The predictive value of radiological diagnosis of UIP by HRCT seems to be 90–100% [12] but a significant minority of patients with histopathological UIP do not fulfil HRCT criteria for UIP [13,14].

Sarcoidosis is a systemic granulomatosis of unknown aetiology that mainly occurs in females in the 20–40 years old age range. It is reported to have a lower incidence among smokers, as well as seasonal and geographical variations [15,16,17,18]. Sarcoidosis features non-caseating giant-cell granulomas in affected organs, mainly the lungs, with variable course ranging from spontaneous regression (Lofgren syndrome) to chronic inflammation and fibrosis [16,19,20]. It may be asymptomatic at onset (up to 30% of patients) with incidental diagnosis by chest X-ray. Different radiological features, including linear opacities, fissure displacement, bronchovascular distortion, bronchiectasis and honeycombing restricted to the upper zones of the lungs, are the classical irreversible changes seen in fibrotic sarcoidosis [21]. There is some evidence that sarcoidosis is related to immune dysfunction and that genetic predisposition may play a role in certain populations [22,23]. Diagnosis of sarcoidosis is confirmed by lung biopsy evidence of non-necrotizing epithelioid granulomas, with or without associated chronic inflammatory infiltrates, in a clinical, laboratory and radiologic context suggesting the disease [15].

Idiopathic pulmonary fibrosis and sarcoidosis are very different diseases in terms of aetiology, epidemiology, risk factors, symptoms and chest imaging. It is not clear whether UIP is the common final pathway of advanced pulmonary fibrosis, or whether CSIPF patients have an overlap of the two conditions. This distinction may have important implications for prognosis and therapy. Recent papers reporting an overlap of sarcoidosis and IPF suggest the existence of a distinct disease which has been called *combined sarcoidosis and idiopathic pulmonary fibrosis* (CSIPF).

The aim of the present study was to systematically review the literature on CSIPF patients. A secondary aim was to analyse and compare Italian cohorts of CSIPF, lone-IPF and fibrotic sarcoidosis patients in order to highlight possible differences and any relationship between these disorders.

## 2. Methods

We used the scoping review protocol [24] and descriptive thematic analysis, detailed below, to review the literature regarding CSIPF. This article conforms to the guidelines of the Scale for Assessment of Narrative Review Articles (SANRA) [25].

### 2.1. Eligibility Criteria

The inclusion criteria were peer-reviewed, empirical or prospective papers (including editorials, commentaries and brief commentaries): (a) with pertinence to the study topic; (b) in English; (c) preferably in journals related to pneumology with full text or abstract; (d) in the form of review, case report, case series, original article or letter to the editor. Studies were excluded if they met any of the following criteria: (a) they were not pertinent to the topic of the study; (b) they were written in a language different from English; (c) the full text of abstracts was not (yet) available.

### 2.2. Information Sources and Search

A systematic search of the literature was conducted in the PubMed online database. The terms entered in our Boolean search syntax were: “combined sarcoidosis and idiopathic pulmonary fibrosis”, “CSIPF”, “sarcoidosis and pulmonary fibrosis”, and “sarcoidosis and usual interstitial pneumonia”. We did not search the grey literature (e.g., official reports from international organizations).

### 2.3. Selection Process

Three independent reviewers (L.B., G.M. and G.N.) screened the abstracts and titles, and ascertained availability of the full texts. If the papers met the eligibility criteria, they were considered. Any disagreement of the reviewers was resolved by consensus.

### 2.4. Data Charting and Items

**Figure 1:**
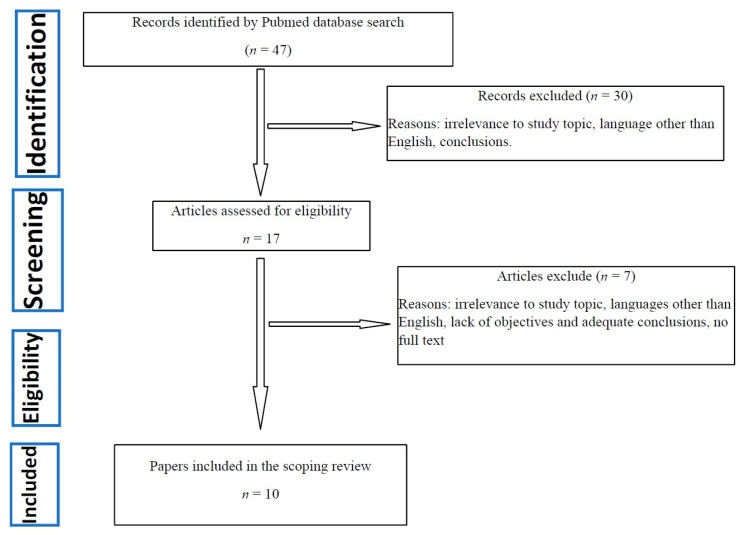
Flowchart of selected manuscripts.

One author (L.B.) extracted formal data items, including publication type, sources, geographies, objectives and main findings; a random of 5% of items were verified by another author (G.N.). Three independent reviewers (L.B, G.M and G.N.) extracted text quotations on either of the following: (1) data collected for each cohort; (2) comparison cohort used to discriminate this possible new disease phenotype. These independent extractions were later paired for qualitative data synthesis, which was also supplemented with a brief summary of each paper, drafted independently by two reviewers. The content of these extractions and the reviewers’ combined summary of each paper were then merged. Figure 1 shows a flowchart of selected articles.

### 2.5. Patient Cohorts

Nine CSIPF patients (88% male, mean age 62.9 ± 10.4 years) seen at the Siena Regional Referral Centre for Sarcoidosis and other Interstitial Lung Diseases and at the Respiratory Diseases Unit, Florence University Hospital, Florence, Italy, in the period 2014–2021, were enrolled retrospectively. Control cases seen in the same period were randomly selected from the database and included 19 lone-IPF patients and 26 patients with fibrotic sarcoidosis. Diagnoses were performed according to international guidelines [6]. Patients with another potential cause of UIP, such as connective tissue diseases (CTD), hypersensitivity pneumonitis (HP) or drug-associated ILD, were excluded. Demographic data, medical history, environmental exposures (occupational and domestic) and prior medication were recorded for all patients. Lung function parameters were recorded at baseline and at one- (T1) and two-year (T2) follow-up. All patients gave their written informed consent to participation in the study, which was approved by our local ethics committee (CEAVSE, Markerlung 17431).

### 2.6. Statistical Analysis

Results are reported as mean ± standard deviation (M ± SD) or medians and interquartile range (median; (IQR)) for continuous variables, as appropriate. The Shapiro–Wilk test was used to check normal distribution of the variables. Non-parametric one-way ANOVA (Kruskal–Wallis test) and Dunn test were used for multiple comparisons. The Chi-squared and Fisher tests were used for categorical variables. Two-dimensional ANOVA (Friedman test) was used to analyse 2 × 2 matrices of variables.

## 3. Results

### 3.1. Descriptive Data and Quality Assessment after SANRA Assessment

The literature search yielded entire texts, of which we selected ten: two comparative studies, one case series, two case reports, three reviews and two comments/editorials. Table 1 shows the results of SANRA. All 30 ratings (3 raters × 10 manuscripts) were used for statistical analysis. The mean sum score across all 10 manuscripts was 9.6 out of 12 possible points (SD 1.7 range 8–11.5, median 9). The highest scores were rated for items 1 and 6 (justification of the article’s importance for the readers and appropriate presentation of data) (mean 1.9), and the lowest for items 3 and 5 (mean 1.4).

### 3.2. Cohort Description

All our CSIPF patients were Caucasian and showed a prevalence of males and ex-smokers. Eight were initially diagnosed with sarcoidosis and one with IPF. Mean age at diagnosis of sarcoidosis was 69.5 ± 8.7 years, which was significantly different from the mean age at diagnosis of patients with fibrotic sarcoidosis (*p* < 0.0001). We did not observe any difference in mean age at diagnosis between patients with lone-IPF and CSIPF (*p* > 0.05). Patients with sarcoidosis were predominantly non-smoker females who were younger than patients in the other groups (*p* < 0.05). Among patients with CSIPF, sarcoidosis was ascertained by histopathology in 8/9 cases. Seven out of nine CSIPF patients (77%) had a family history of lung fibrosis compared to only two patients with IPF. Two out of nine CSIPF patients (23%) showed extrapulmonary manifestations of sarcoidosis (eye = 2, spleen = 1) compared to nine (38%) of the fibrotic sarcoidosis group (spleen = 4, skin = 3, liver = 2). The main comorbidities of lone-IPF and CSIPF patients were gastroesophageal reflux disease (GERD) and arterial hypertension, while those of the sarcoidosis group were infectious diseases and other lung disorders. All patients with lone-IPF and CSIPF underwent antifibrotic therapies (Table 2).

### 3.3. Lung Function Tests

**Figure 2:**
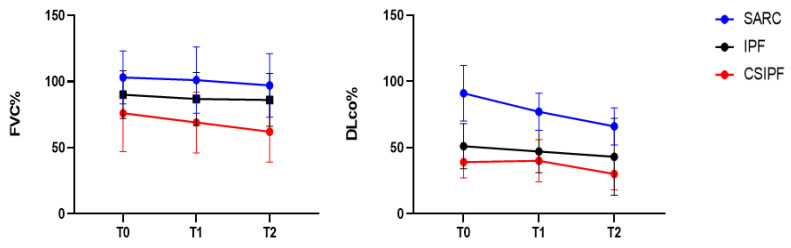
FVC and DLco percentages at diagnosis (T0) and at one- (T1) and two-year (T2) follow-up.

On average, functional assessment at baseline showed mild to moderate restrictive impairment of lung volumes in lone-IPF and CSIPF patients (%FVC 90 ± 18 and 76 ± 29, respectively), associated with moderate-to-severe reduction in DLco percentages (51 ± 17 and 39 ± 12, respectively); however, no statistically significant differences emerged at baseline between these two groups. The fibrotic sarcoidosis group differed greatly from other two groups of patients; specifically, no restrictive impairment was found with respect to the CSIPF group: FVC% 103 ± 20 vs. 76 ± 29 (*p* = 0.02), respectively; mean DLco% 89 ± 17 vs. 39 ± 12 (*p* = 0.02), respectively. Interestingly, at the two-year follow-up, a statistically significant decrease in FVC% emerged for CSIPF patients (−14%, *p* = 0.0053) and in DLco percentages for the fibrotic sarcoidosis cohort (−11%, *p* = 0.044) (Figure 2).

## 4. Discussion

In recent years, a disease phenotype consisting in an overlap between sarcoidosis and IPF, known as CSIPF, has been postulated. The present case series included nine patients with CSIPF. In line with the literature, these patients showed different features in terms of age, occupational exposure and granuloma localization [26,27]. They showed faster functional deterioration during two years of follow-up than patients with lone-IPF or fibrotic sarcoidosis. It is not yet known whether this clinical condition is an evolution of sarcoidosis or a real disease phenotype. In fact, up to 20% of sarcoidosis patients develop fibrotic lung disease, whereby granulomatous inflammation evolves into pulmonary fibrosis with a high mortality rate [27]. From the functional point of view, patients with advanced fibrotic sarcoidosis show a combination of airway dysfunction (obstruction) superimposed on the more common restrictive dysfunction [13,14]. Treatment, usually with corticosteroids, may be effective for fibrotic sarcoidosis, but the fibrosis may not improve with any treatment, making lung transplant the only feasible option [28]. Two recent papers reported a cohort of CSIPF patients, prior to which there were various case reports. In 2006, Nobata K reported the first case with typical pulmonary sarcoidosis, characterized by an increase in BAL lymphocyte percentages and CD4/CD8 ratio, biopsy evidence of non-caseating epithelioid cell granulomas and high concentrations of sarcoid-related biomarkers. After two years, it was difficult to differentiate pulmonary sarcoidosis from UIP, because inflammatory and fibrotic features were both found [29]. In 2012, Tachibana K et al. described similar findings in another case of sarcoidosis with lower lung dominant reticular shadows. UIP pattern developed after 3 years, with HRCT evidence of honeycombing and extensive alveolar damage, later progressing to acute exacerbation. An overlap between sarcoidosis and UIP was suggested [30]. The first comparative study was reported in 2013 by Xu L et al. [31]. Nine histological sections from lung explants with end-stage sarcoidosis were studied and fibrotic and active granulomatous patterns, both very distinct from those seen in UIP, were observed. Three showed areas of honeycombing with surrounding scarring. Unlike UIP, this honeycombing was predominantly central with prominent bronchiectasis [31].

Collins BF et al. described a cohort of 25 patients who manifested combined clinical and radiological features of IPF and sarcoidosis (CSIPF). Clinical course and mortality rate were similar in groups of patients with lone-IPF and CSIPF. The authors concluded that sarcoidosis and IPF co-existed in these patients but that their onset occurred at different times [32]. Two subsequent commentaries highlighted the importance of further longitudinal studies and a prospective cohort [33].

In 2019, Bianchi et al. described a case series of four patients with overlapping sarcoidosis and idiopathic pulmonary fibrosis. This study confirmed that this rare association is more frequent in males with a documented family history of ILD and that the sarcoidosis prevalently affected the lungs [34].

Findings in our cohort confirmed the previous literature. In particular, our patients were prevalently male ex-smokers, with an age intermediate between those of the sarcoidosis and lone-IPF groups. Occupational exposure seemed to play a crucial role in this cohort. The main comorbidities were similar to those of the lone-IPF group and most cases did not show extrapulmonary localizations of granulomas. Interestingly, lung function deteriorated during the two years of follow-up, despite that fact that all CSIPF patients were on antifibrotic treatments. This result suggests that these patients are at high risk of rapid deterioration and lung damage.

## 5. Conclusions

CSIPF showed different features in terms of age, occupational exposure and localization of granulomas. Faster functional deterioration than in lone-IPF and fibrotic sarcoidosis patients was observed during the two years of follow-up. These patients have a worse prognosis, making it opportune to investigate a large international cohort of CSIPF patients.

## Figures and Tables

**Table 1:** SANRA Quality assessment of selected manuscript.

No.	Title and Authors	Justification of the Article’s Importance for the Readership	Statement of Concrete Aims or Formulation of Questions	Description of the Literature Search	Referencing	Scientific Reasoning	Appropriate Presentation of Data	Total Score
1	Bianchi, F., Piccioli, C., Rosi, E., Carobene, L., Spina, D., Mazzei, M. A., Bartolucci, M., Moroni, C., Novelli, L., Rottoli, P., & Bargagli, E. (2019). Combined sarcoidosis and idiopathic pulmonary fibrosis (CSIPF): A novel disease phenotype? Respiratory medicine, 160, 105650.	2	1	2	2	1	2	10
2	Morgenthau A. S. (2018). Combined sarcoidosis and idiopathic pulmonary fibrosis (CSIPF): Genuine disease entity, obscure clinical phenotype or diagnostic red herring? Respiratory medicine, 144S, S3–S4.	2	2	2	2	2	2	12
3	Collins, B. F., McClelland, R. L., Ho, L. A., Mikacenic, C. R., Hayes, J., Spada, C., & Raghu, G. (2018). Sarcoidosis and IPF in the same patient-a coincidence, an association or a phenotype? Respiratory medicine, 144S, S20–S27.	2	2	2	2	2	2	12
4	Tachibana, K., Arai, T., Kagawa, T., Minomo, S., Akira, M., Kitaichi, M., & Inoue, Y. (2012). A case of combined sarcoidosis and usual interstitial pneumonia. Internal medicine (Tokyo, Japan), 51(14), 1893–1897	2	2	1	2	1	1	9
5	Collins, B. F., & Raghu, G. (2019). Sarcoidosis and idiopathic pulmonary fibrosis: The same tale or a tale of two diseases in one. Respiratory medicine, 160, 105668.	2	2	2	2	2	2	12
6	Shigemitsu, H., & Azuma, A. (2011). Sarcoidosis and interstitial pulmonary fibrosis; two distinct disorders or two ends of the same spectrum. Current opinion in pulmonary medicine, 17(5), 303–307	2	1	1	2	2	2	8
7	Patterson, K. C., & Strek, M. E. (2013). Pulmonary fibrosis in sarcoidosis. Clinical features and outcomes. Annals of the American Thoracic Society, 10(4), 362–370.	1	2	1	1	1	2	8
8	Teirstein, A. T., & Morgenthau, A. S. (2009). “End-stage” pulmonary fibrosis in sarcoidosis. The Mount Sinai journal of medicine, New York, 76(1), 30–36.	2	1	1	1	1	2	8
9	Xu, L., Kligerman, S., & Burke, A. (2013). End-stage sarcoid lung disease is distinct from usual interstitial pneumonia. The American journal of surgical pathology, 37(4), 593–600.	2	1	1	1	1	2	8
10	Nobata, K., Kasai, T., Fujimura, M., Mizuguchi, M., Nishi, K., Ishiura, Y., Yasui, M., & Nakao, S. (2006). Pulmonary sarcoidosis with usual interstitial pneumonia distributed predominantly in the lower lung fields. Internal medicine (Tokyo, Japan), 45(6), 359–362	2	2	1	1	1	2	9

**Table 2:** Demographic, historical and imaging characteristics of IPF, CSIPF and lone-IPF cohorts.

Characteristics	CSIPF (*n* = 9)	Lone-IPF (*n* = 19)	Stage 4 Sarcoidosis (*n* = 26)	*p*-Values
Sex (m/f)	8/1	(15/4)	(7/19)	0.0001
Caucasian (*n*)	8	19	25	ns
Smoking (current/never/former)	0/2/7	0/4/15	1/16/9	0.01
Age at sarcoidosis diagnosis (CSIPF diagnosis of granulomatous disease) (mean ± S.D)	69.5 ± 8.7	na	42.8 ± 12.4	0.0001
Age at IPF diagnosis (mean ± S.D)	62.9 ± 10.4	72 ± 7.1	na	0.06
Family history (*n*)	7	2	0	0.0001
Occupational exposure (yes/no)	2/7	14/5	15/11	0.01
Main comorbidities				na
● GERD	2	5	0	
● PH	1	1	2	
● AH	3	3	2	
● Infectious diseases	2	1	3	
● Other lung disorders	1	2	7	
Extrapulmonary localization (yes/no)	2/7	na	10/16	0.03
Antifibrotic Therapy			na	na
● Pirfenidone	6	10		
● Nintedanib	3	9		
HRCT evidence of UIP				na
● Consistent	6	16	0	
● Inconsistent	0	0	26	
● Possible	2	3	0	

## Data Availability

The datasets generated during and/or analysed during the current study are available from the corresponding author on reasonable request.

## Abbreviations

Combined sarcoidosis and idiopathic pulmonary fibrosis (CSIPF), forced vital capacity (FVC), diffusing capacity of the lung for carbon monoxide (DLco), Forced Expiratory Volume in the 1st second (FEV1).
